# Temporal trends in clinical features of patients with primary aldosteronism over 20 years

**DOI:** 10.1038/s41440-024-01703-w

**Published:** 2024-05-17

**Authors:** Seung Shin Park, Chang Ho Ahn, Sang Wan Kim, Jung-Min Koh, Seung Hun Lee, Jung Hee Kim

**Affiliations:** 1 Department of Internal Medicine, Seoul National University College of Medicine, Seoul, Korea; 2 Department of Internal medicine, Seoul National University Hospital, Seoul, Korea; 3 Department of Internal medicine, Seoul National University Bundang Hospital, Sungnam, Korea; 4 Division of Endocrinology and Metabolism, Department of Internal Medicine, Boramae Medical Center, Seoul, Korea; 5 Division of Endocrinology and Metabolism, Department of Medicine, Asan Medical Center, University of Ulsan College of Medicine, Seoul, Korea

**Keywords:** Adrenal incidentaloma, Aldosterone, Aldosterone producing adenoma, Bilateral adrenal hyperplasia, Primary aldosteronism

## Abstract

Primary aldosteronism (PA) accounts for approximately 5-10% of hypertension cases. Over the past 20 years, the reported incidence of PA has increased due to widespread screening for secondary hypertension and imaging studies. We aimed to evaluate the temporal trends in the clinical characteristics and subtypes of PA. A total of 1064 patients with PA in two tertiary hospitals between 2000 and 2021 were categorized into three groups according to the year of diagnosis: 2000–2009, 2010–2015, and 2016–2021. The clinical characteristics of the patients over the three time periods were compared using a trend analysis. The age at diagnosis and sex of patients with PA did not change over 20 years. The proportion of patients with bilateral hyperaldosteronism (BHA) increased (11%, 25%, and 40%, *P* for trend <0.001). The proportion of hypokalemia (87%, 61%, and 40%) and plasma aldosterone concentration (36.0, 30.8, and 26.6 ng/dL) decreased (all *P* for trend <0.001). There was a trend toward an increased proportion of incidentally detected patients compared to clinically symptomatic patients (36%, 55%, and 61%, *P* for trend <0.001). The concordance rate of imaging and adrenal venous sampling results decreased (91%, 70%, and 57% *P* for trend <0.001). However, the proportion of patients with resistant hypertension and comorbidities did not differ. In conclusion, among patients with PA, patients with BHA and incidental detection have increased over 20 years, and more patients are likely to present with milder clinical symptoms and biochemical profiles.

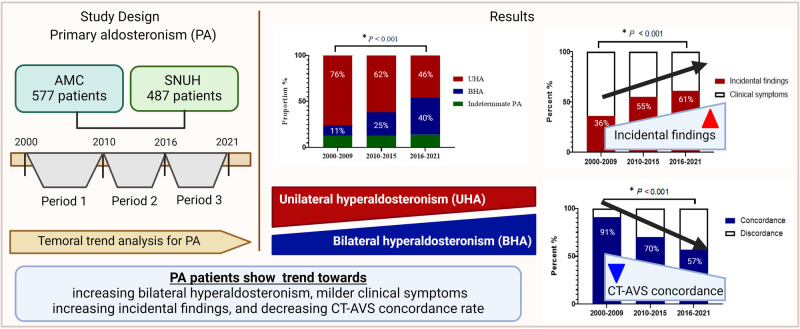

## Introduction

Primary aldosteronism (PA) is caused by the autonomous secretion of aldosterone in the adrenal gland [1]. It is considered the most common cause of secondary hypertension (HTN), accounting for approximately 5%–10% of all HTN cases [1–3]. Patients with PA are at a higher risk for cardiovascular diseases, atrial fibrillation, stroke, type 2 diabetes, metabolic syndrome, and mortality than those with essential hypertension [4–7]. Therefore, early diagnosis and treatment of PA have been emphasized [8].

PA has two main subtypes: aldosterone-producing adenoma (APA) and bilateral adrenal hyperplasia (BAH). Although the clinical characteristics and biochemical profiles of these two subtypes are not completely distinct, some differences exist between them. Severe aldosterone excess and hypokalemia are characteristic features of APA. In contrast, relative normokalemia and mild-to-moderate aldosterone excess are characteristics of BAH [9].

The reported incidence of PA has increased over the decades [10], chiefly due to frequent routine check-ups and increased screening for PA among HTN patients. With the increase in PA incidence, our knowledge regarding PA characteristics has also evolved. Few studies have shown secular trends of clinical characteristics of PA over the past years [11, 12]. Trends towards decreasing unilateral PA and diagnosis of milder forms of PA have been observed between 2008 and 2016 [11]. However, these trends have been investigated over a relatively short duration (approximately 9 years). In addition, because the enrolled patients were mainly Europeans, it is difficult to say if similar trends would be observed in Asian countries owing to differences in the genetic background for PA [13].

The reported incidence of adrenal incidentaloma has increased with the widespread use of imaging techniques and frequent routine check-ups. Since hormone tests are recommended to assess the functionality of adrenal incidentalomas, an increase in adrenal incidentaloma incidence has also led to an increase in the detection of PA. Few studies have compared the clinical characteristics of patients with incidental findings and those with clinical symptoms. Lindsay et al. observed no significant difference in the clinical and demographic characteristics of patients according to the method of discovery (i.e., incidental PA and nonincidental with clinical symptoms) [14]. However, their study targeted only patients who underwent surgical intervention. Thus, it remains unclear if the clinical or biochemical characteristics of patients with PA differ according to the method of discovery.

We aimed to evaluate the temporal trends in the clinical characteristics and subtypes of PA. We also aimed to evaluate the clinical and biochemical characteristics of PA according to the method of discovery.

Point of view

- Clinical relevance
  There are trends toward increasing the proportions of bilateral hyperaldosteronism, incidental discoveries, and clinically and biochemically milder forms of primary aldosteronism.
- Future direction
  A further study is required to assess the impact of these trends on the patients’ treatment outcomes.
- Consideration for the Asian population
  Differences in gene variants between European and Asian populations, such as the higher prevalence of KCNJ5 mutation in Asia, could impact the temporal trends of primary aldosteronism.

## Methods

### Study subjects

This study is part of The Korean Primary Aldosterone Study (KPAS), an ongoing cohort study of PA [9]. A total of 1,062 PA patients were consecutively enrolled from two tertiary hospitals in Korea, Seoul National University Hospital (SNHU, *n* = 487) and Asan Medical Center (AMC, *n* = 577), between 2000 and 2021. The PA patients were categorized into three groups according to the year of diagnosis: period 1 (2000–2009), period 2 (2010–2015), and period 3 (2016–2021). This study was approved by the Institutional Review Boards of SNUH (No. 2204-155-1320) and AMC (No. 2022-1496). It was conducted in compliance with the Declaration of Helsinki guidelines. The requirement for written informed consent was waived owing to the retrospective nature of this study.

### Diagnosis and subtyping of PA

Confirmatory testing was performed in patients with an aldosterone/renin ratio (ARR) ≥ 20 and plasma aldosterone concentration (PAC) > 15 ng/dL. We diagnosed PA in the case of PAC > 10 ng/dL after the saline infusion test in a recumbent position [1]. Prior to either screening testing using ARR measurement or confirmatory testing, diuretics/mineralocorticoid receptor antagonists and beta-adrenergic receptor blockers were changed to calcium channel or alpha-adrenergic receptor blockers for ≥6 and ≥2 weeks. However, PA was diagnosed without a confirmatory test in those patients with spontaneous hypokalemia, a plasma renin activity (PRA) below the detection limit, or a PAC > 20 ng/dL [1].

Subtypes of PA were categorized as unilateral hyperaldosteronism (UHA), bilateral hyperaldosteronism (BHA), and indeterminate type based on abdominal computed tomography (CT) findings and adrenal venous sampling (AVS) results under adrenocorticotropic hormone stimulation [15, 16]. AVS was recommended when surgical treatment was feasible and desired by the patient. AVS was not performed when surgical treatment was not feasible or if there was renal dysfunction. The selectivity index (SI, adrenal vein cortisol concentration/inferior vena cava cortisol concentration) >3 was defined as successful AVS. The lateralization index (LI) was calculated by dividing the aldosterone-to-cortisol ratio on the dominant side by that on the non-dominant side, and an LI ≥ 4 was considered to indicate lateralized aldosteronism. UHA was defined when aldosterone secretion appeared lateralized based on AVS results [15, 16]. BHA was defined as a successful AVS result showing an LI < 3, regardless of the adrenal nodule on abdominal CT findings. The indeterminate PA subtype was defined as AVS failure or 3 ≤ LI < 4 on AVS results or no AVS data. The Contralateral ratio index (CI) was calculated by dividing the aldosterone-to-cortisol ratio on the non-dominant side by that on the inferior vena cava. The contralateral ratio index (CI) was calculated by dividing the aldosterone-to-cortisol ratio on the non-dominant side by that on the inferior vena cava. Contralateral suppression with a CI < 1 can be used as indicative of UHA on the opposite side in cases of indeterminate PA subtype (*n* = 162) [17]. Fifteen cases out of the 162 with an indeterminate PA subtype were recategorized as UHA. Concordance between CT and AVS findings was established when a unilateral adrenal nodule (>0.7 cm) was identified on CT scans with AVS confirmation of lateralization to the same side, or when CT scans showed only normal-appearing adrenals and the AVS was indicative of BHA.

### Clinical and biochemical parameters

Anthropometric and clinical data, including age, sex, body mass index (BMI), and blood pressure were collected. BMI was calculated by dividing weight (kg) by height (m^2^). The defined daily dose (DDD) of antihypertensive medication was based on the World Health Organization Anatomical Therapeutic Chemical/Defined Daily Dose (DDD) Index (https://www.whocc.no/atc_ddd_index).

PAC and plasma renin activity (PRA) were measured by radioimmunoassay (RIA) using the SPAC-S Aldosterone kit (TFB Inc., Tokyo, Japan) for PAC and PRA RIA kits (TFB Inc.) for PRA at SNUH before 2011 or Renin RIA beads (TFB Inc.) for PRA at SNUH after 2011 and AMC. The coefficients of variation for the intra- and inter-assays were 5% and 10%, respectively. The measurements of PAC by RIA were not standardized by liquid chromatography-tandem mass spectrometer measurements. A Cobas 8000 ISE analyzer (Roche Diagnostics, Mannheim, Germany) and a Roche ISE Standard Low/High (Roche Diagnostics) ion-selective electrode were used to measure serum potassium concentration.

### Definition of clinical parameters

Hypokalemia was defined as a serum potassium level of <3.5 mEq/L or the use of a potassium supplement. HTN was defined as systolic blood pressure (SBP) > 140 mmHg, diastolic blood pressure (DBP) > 90 mmHg, or ongoing intake of antihypertensive drugs. Diabetes mellitus was defined as having hemoglobin A1c levels ≥6.5%, or fasting blood glucose levels ≥126 mg/dL, confirmed on two or more occasions, or by ongoing treatment with oral hypoglycemic agents or insulin. Chronic kidney disease (CKD) ≥stage 3 was considered when the estimated glomerular filtration rate was less than 60 mL/min/1.73 m^2^. Coronary artery disease (CAD) was defined as the presence of unstable angina or a history of percutaneous coronary intervention or coronary artery bypass graft surgery. Physician-adjudicated cerebrovascular accidents (CVA), including ischemic or hemorrhagic stroke, and atrial fibrillation were considered. Incidental findings were defined if adrenal nodules or hyperplasia were incidentally detected during routine health check-ups or workups for other medical conditions. On the other hand, in cases where primary aldosteronism is diagnosed during the evaluation of symptoms related to conditions like hypertension, it is defined as being discovered based on clinical symptoms.

### Statistical analysis

Continuous variables are presented as mean ± standard deviation. For categorical variables, data are presented as numbers (percentages, %). The Jonckheere-Terpstra trend test was used to compare continuous variables among the three time periods (2000–2011, 2011–2015, and 2016–2021), and the Cochran-Armitage trend test was used for categorical variables. In addition, Student’s *t*-test was used to compare continuous variables according to the detection methods, while the Chi-square test was used for categorical variables. Statistical significance was set at *P* < 0.05. SPSS, version 26.0 (IBM, Armonk, NY, USA) and R version 4.1.2 (Foundation for Statistical Computing, Vienna, Austria) were used for the statistical analysis.

## Results

A total of 1064 PA patients were identified between 2000 and 2021. The mean age of the PA patients was 52.3 ± 11.7 years, and 530 (50%) patients were female. We categorized PA patients into three groups according to the year at diagnosis: 2000–2009 (1), 2010–2015 (2), and 2016–2021 (3). Table 1 shows the time trends in the clinical characteristics of patients with PA according to the time period of diagnosis. The number of PA patients increased from 129 in 2000-2009 to 660 patients in 2016–2021. There was no significant trend in age at diagnosis or sex distribution among the three groups. However, the BMI of patients showed trends of increase over time (*P* for trend <0.001). Furthermore, we had similar findings when we reclassified our PA patients into three diagnostic periods of equal length (2000–2007: 78 patients; 2008–2014: 276 patients, and 2015–2021: 710 patients; Supplementary Table S1).

**Table 1:** Trends in the clinical characteristics of PA patients according to the period of diagnosis

	2000-2009 (*n* = 129)	2010-2015 (*n* = 275)	2016-2021 (*n* = 660)	Total (*n* = 1064)	*P* for trend
Number of PA patients per year	12.9	45.8	110.0	48.4	
Age (years)	50.0 ± 12.9	52.5 ± 10.6	52.7 ± 11.8	52.3 ± 11.7	0.103
Female (*n*, %)	62 (48.1%)	137 (49.8%)	331 (50.2%)	530 (49.8%)	0.694
Height (cm)	162.9 ± 8.2	163.7 ± 8.4	164.4 ± 8.4	164.0 ± 8.4	0.056
Weight (kg)	66.1 ± 11.0	67.6 ± 13.3	70.5 ± 14.7	69.2 ± 14.0	**<0.001**
BMI (kg/m^2^)	24.8 ± 3.3	25.0 ± 3.5	25.9 ± 4.0	25.6 ± 3.8	**<0.001**
SBP (mmHg)	149.8 ± 25.0	142.1 ± 19.0	141.3 ± 17.8	142.5 ± 19.3	**0.028**
DBP (mmHg)	92.9 ± 17.8	88.1 ± 13.3	87.7 ± 11.5	88.4 ± 13.0	**0.036**
Subtype					
UHA	98 (76.0%)	171 (62.2%)	301 (45.6%)	570 (53.6%)	**<0.001**
UHA with ipsilateral nodule	92 (71.3%)	140 (50.9%)	233 (35.4%)	465 (43.7%)	**<0.001**
UHA with contralateral nodule	1 (0.8%)	7 (2.5%)	23 (3.5%)	31 (2.9%)	0.089
UHA with bilateral nodule	4 (3.1%)	11 (4.0%)	30 (4.6%)	45 (4.2%)	0.439
UHA without nodule	1 (0.8%)	13 (4.7%)	15 (2.3%)	29 (2.7%)	0.902
BHA	14 (10.9%)	69 (25.1%)	264 (40.0%)	347 (32.6%)	**<0.001**
BHA with bilateral nodule	1 (0.8%)	8 (2.9%)	20 (3.0%)	29 (2.7%)	0.223
BHA with unilateral nodule	8 (6.2%)	49 (17.8%)	179 (27.2%)	236 (22.2%)	**<0.001**
BHA without nodule	5 (3.9%)	12 (4.4%)	64 (9.7%)	81 (7.6%)	**0.002**
Indeterminate PA^a^	17 (13.2%)	35 (12.7%)	95 (14.4%)	147 (13.8%)	0.557
Nodule size (cm)	1.7 ± 0.7	1.6 ± 0.8	1.6 ± 0.8	1.6 ± 0.8	0.391
PAC (ng/dL)	36.0 [26.4;51.3]	30.8 [22.5;44.9]	26.6 [21.2;37.8]	28.8 [21.9;41.5]	**<0.001**
PRA (ng/mL/hr)	0.20 [0.10;0.40]	0.20 [0.10;0.42]	0.24 [0.20;0.46]	0.20 [0.10;0.45]	**<0.001**
ARR (ng/dL per ng/mL/h)	207.0 [85.9;393.5]	142.0 [71.8;314.0]	106.3 [53.9;205.0]	119.0 [59.3;251.4]	**<0.001**
Lowest K (mmol/L)	3.1 [2.8; 3.4]	3.5 [3.0; 4.0]	3.8 [3.2; 4.1]	3.6 [3.0; 4.0]	**<0.001**
Hypokalemia^b^ (*n*, %)	112 (86.8%)	166 (60.8%)	266 (40.4%)	544 (51.3%)	**<0.001**
HTN (*n*, %)	128 (99.2%)	269 (97.8%)	611 (92.6%)	1008 (94.7%)	**<0.001**
Duration of HTN (years)	6.5 ± 6.0	7.7 ± 6.9	7.2 ± 8.0	7.3 ± 7.5	0.126
Antihypertensive drug, DDD	2.1 ± 1.8	2.0 ± 1.6	2.1 ± 1.9	2.1 ± 1.8	0.848
Antihypertensive drug, DDD ≥ 3 (*n*, %)	36 (28.1%)	78 (29.4%)	185 (28.7%)	299 (28.8%)	0.994
DM (*n*, %)	15 (11.6%)	38 (13.8%)	105 (15.9%)	158 (14.9%)	0.168
CKD ≥ stage 3^c^ (*n*, %)	11 (8.5%)	18 (6.7%)	39 (6.0%)	68 (6.5%)	0.296
CAD^d^ (N, %)	7 (5.4%)	24 (8.7%)	52 (7.9%)	83 (7.8%)	0.543
Atrial fibrillation (*n*, %)	4 (3.1%)	3 (1.1%)	8 (1.2%)	15 (1.4%)	0.200
CVA (*n*, %)	10 (7.8%)	22 (8.0%)	38 (5.8%)	70 (6.6%)	0.229

*ARR* aldosterone renin ratio, *BHA* bilateral hyperaldosteronism, *BMI* body mass index, *CAD* coronary artery disease, *CKD* chronic kidney disease, *CVA* cerebrovascular disease, *DBP* diastolic blood pressure, *DM* diabetes mellitus, *DDD* daily drug dosage, *HTn* hypertension, *K* potassium, *PA* primary aldosteronism, *PAC* plasma aldosterone concentration, *PRA* plasma renin activity, *OSA* obstructive sleep apnea, *SBP* systolic blood pressure, *UHA* unilateral hyperaldosteronism. Data are shown as mean ± standard deviation for continuous variables, *N*(%) for categorical variables. Jonckheere-Terpstra trend test was used for continuous variables and Cochran-Armitage trend test was used for categorical variables. *P* values < 0.05, indicating statistical significance, are highlighted in bold

^a^To determine the PA subtype, we used the lateralization index (LI), which was calculated by dividing aldosterone to cortisol ratio on the dominant side by that on the non-dominant side. The intermediate subtype was defined as an LI with intermediate values (3 ≤ LI < 4)

^b^Hypokalemia was defined if the serum potassium was <3.5 mEq/L or a patient was taking a potassium supplement

^c^CKD ≥ stage 3 was defined as the estimated glomerular filtration rate (eGFR) < 60 mL/min/1.73 m^2^

^d^CAD was defined if patients had a history of percutaneous coronary intervention (PCI), coronary artery bypass graft (CABG) surgery, or unstable angina

**Fig. 1:**
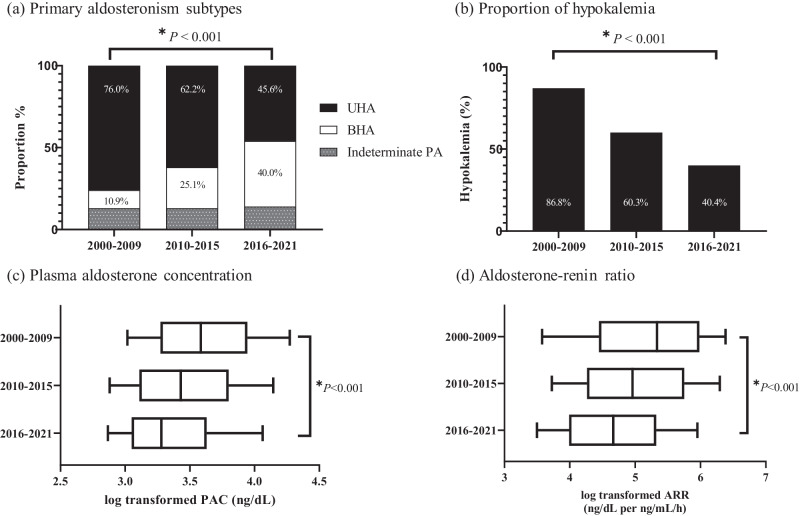
Temporal trend of clinical characteristics in patients with primary aldosteronism. **a** Primary aldosteronism subtypes. **b** Proportion of hypokalemia. **c** Plasma aldosterone concentration. **d** Aldosterone-renin ratio. UHA unilateral hyperaldosteronism, BHA bilateral hyperaldosteronism, PAC plasma aldosterone concentration, ARR aldosterone renin ratio

Significant changes were observed in the proportion of PA subtypes. The proportions of UHA and BHA showed opposite trends over time. UHA showed a gradual decrease, whereas BHA showed a gradual increase over the 20 years (76.0%, 62.2%, and 45.6%, for UHA; 10.9%, 25.1%, and 40.1% for BHA, all *P* for trend <0.001) (Table 1, Fig. 1a). Intriguingly, the proportion of indeterminate PA cases showed a similar trend from time period 1 through 3 (13.2%, 12.7%, and 14.4%, *P* for trend 0.557). Significant changes in the proportion of PA subtypes were also observed in the 2000–2007, 2008–2014, and 2015-2021 periods (Supplementary Table S1).

PA severity attenuated from periods 1 to 3. BP and the prevalence of HTN decreased (all *P* for trend <0.050), although the duration of HTN, DDD of antihypertensive drugs, and prevalence of resistant HTN did not change over time. The proportion of patients with hypokalemia significantly diminished over 20 years (86.8%, 60.8%, and 40.4%, *P* for trend <0.001) (Fig. 1b). PAC and ARR, indicators of PA severity, also decreased from period 1 to 3 (all *P* for trend <0.001) (Fig. 1c, d). Comorbidities, including CKD, CAD, atrial fibrillation, and CVA, did not show significant trends among the three time periods.

We further analyzed the temporal trends of the subtype-specific clinical characteristics according to the three time periods (Table 2). In patients with BHA, the proportion of females tended to increase in recent years, while there was no sex-based difference among patients with UHA. In patients with UHA, the lowest serum potassium levels increased and the proportion of patients with hypokalemia and HTN decreased (all *P* for trend <0.05). A similar tendency of the proportion of patients with hypokalemia and HTN was observed in patients with BHA. PAC was similar between the three time periods in patients with UHA, while it was significantly decreased in patients with BHA. PRA was significantly increased in patients with UHA, while it was similar in patients with BHA. ARR tended to decrease in patients with UHA and BHA (*P* for trend = 0.010 for UHA, *P* for trend=0.015 for BHA). The proportion of incidental findings in UHA was more common in period 2 (52.0%) and period 3 (51.5%) than in period 1 (34.7%, *P* for trend = 0.015). The proportion of incidental findings in BHA was also more common in period 2 (68.1%) and period 3 (63.6%) than in period 1 (28.6%), albeit the trend was not statistically significant (*P* for trend=0.193).

**Table 2:** Trends in clinical characteristics of the PA patients with (**a**) UHA and (**b**) BHA according to the period of diagnosis

(a)					
	2000-2009 (*n* = 98)	2010-2015 (*n* = 171)	2016-2021 (*n* = 301)	Total (*n* = 570)	*P* for trend
Age (years)	49.2 ± 12.7	51.7 ± 10.6	50.2 ± 11.1	50.5 ± 11.3	0.927
Female (*n*, %)	49 (50.0%)	98 (57.3%)	150 (49.8%)	297 (52.1%)	0.597
Height (cm)	162.9 ± 8.4	163.2 ± 8.1	164.4 ± 8.2	163.8 ± 8.2	0.058
Weight (kg)	66.0 ± 10.6	65.4 ± 12.3	69.0 ± 14.9	67.4 ± 13.6	**0.042**
BMI (kg/m^2^)	24.8 ± 3.2	24.4 ± 3.3	25.3 ± 3.9	25.0 ± 3.6	0.109
SBP (mmHg)	150.2 ± 23.9	142.8 ± 19.1	142.5 ± 18.0	143.9 ± 19.7	0.107
DBP (mmHg)	93.4 ± 16.8	89.2 ± 13.3	89.0 ± 11.8	89.8 ± 13.3	0.103
PAC (ng/dL)	38.8 [27.2;55.3]	34.1 [24.3;51.5]	33.8 [24.3;51.1]	35.3 [24.5;51.9]	0.139
PRA (ng/mL/h)	0.1 [0.1; 0.4]	0.2 [0.1; 0.3]	0.2 [0.1; 0.4]	0.2 [0.1; 0.3]	**0.002**
ARR (ng/dL per ng/mL/h)	245.0 [96.0;457.0]	178.6 [94.9;383.6]	165.2 [84.2;297.8]	182.0 [87.1;354.0]	**0.010**
Lowest K (mmol/L)	3.0 [2.8; 3.3]	3.1 [2.8; 3.7]	3.4 [3.0; 3.9]	3.3 [2.9; 3.8]	**<0.001**
Hypokalemia^b^ (*n*,%)	93 (94.9%)	133 (77.8%)	187 (62.1%)	413 (72.5%)	**<0.001**
HTN (*n*, %)	97 (99.0%)	167 (97.7%)	285 (94.7%)	549 (96.3%)	**0.027**
Incidental findings (*n*, %)	34 (34.7%)	89 (52.0%)	155 (51.5%)	278 (48.8%)	**0.015**

(b)					
	2000-2009 (*n* = 14)	2010-2015 (*n* = 69)	2016–2021 (*n* = 264)	Total (*n* = 347)	*P* for trend
Age (years)	47.1 ± 15.0	53.1 ± 10.4	54.0 ± 12.2	53.5 ± 12.0	0.303
Female (*n*, %)	5 (35.7%)	25 (36.2%)	135 (51.1%)	165 (47.6%)	**0.024**
Height (cm)	163.9 ± 7.9	164.3 ± 8.5	164.6 ± 8.7	164.5 ± 8.6	0.536
Weight (kg)	66.5 ± 12.2	71.3 ± 14.7	72.6 ± 14.5	72.1 ± 14.4	0.233
BMI (kg/m^2^)	24.3 ± 3.7	26.2 ± 3.8	26.7 ± 4.1	26.5 ± 4.0	0.206
SBP (mmHg)	163.2 ± 35.5	141.6 ± 17.0	140.5 ± 18.0	141.7 ± 19.3	0.251
DBP (mmHg)	100.8 ± 26.0	86.1 ± 12.5	87.0 ± 11.3	87.4 ± 12.7	0.784
PAC (ng/dL)	30.6 [26.5;47.3]	26.8 [21.3;34.8]	23.8 [20.0;29.7]	24.6 [20.2;30.6]	**0.003**
PRA (ng/mL/hr)	0.2 [0.1; 0.4]	0.2 [0.2; 0.5]	0.3 [0.2; 0.5]	0.3 [0.2; 0.5]	0.137
ARR (ng/dL per ng/mL/h)	106.8 [70.0;338.0]	89.7 [54.5;187.5]	77.0 [44.5;127.0]	80.1 [46.7;138.2]	**0.015**
Lowest K (mmol/L)	3.3 [2.9; 3.7]	4.0 [3.7; 4.2]	3.9 [3.6; 4.2]	3.9 [3.6; 4.2]	0.421
Hypokalemia^b^ (*n*,%)	8 (57.1%)	22 (31.9%)	57 (21.7%)	87 (25.1%)	**0.002**
HTN (*n*, %)	14 (100.0%)	69 (100.0%)	239 (90.5%)	322 (92.8%)	**0.006**
Incidental findings (*n*, %)	4 (28.6%)	47 (68.1%)	168 (63.6%)	219 (63.1%)	0.193

*ARR* aldosterone renin ratio, *BHA* bilateral hyperaldosteronism, *BMI* body mass index, *DBP* diastolic blood pressure, *K* potassium, *PA* primary aldosteronism, *PAC* plasma aldosterone concentration, *PRA* plasma renin activity, *SBP* systolic blood pressure, *UHA* unilateral hyperaldosteronism. *P* values < 0.05, indicating statistical significance, are highlighted in bold

Data are shown as a mean ± standard deviation for continuous variables and *n* (%) for categorical variables. A Jonckheere-Terpstra trend test was used for continuous variables and a Cochran-Armitage trend test for categorical variables

**Fig. 2:**
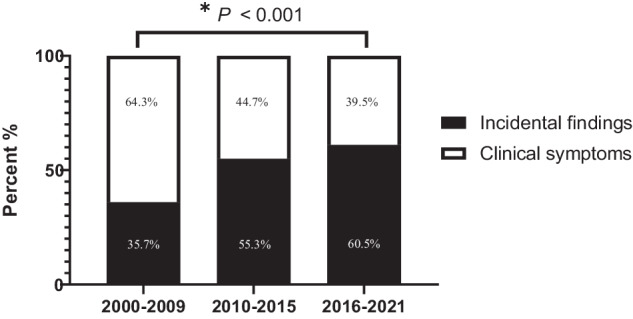
Temporal trend of method of discovery in patients with primary aldosteronism

The method of discovery also changed. The proportion of PA patients diagnosed following incidental findings increased (35.7%, 55.3%, and 6%, *P* for trend <0.001). In contrast, the proportion of PA patients diagnosed based on clinical symptoms declined from time period 1 to 3 (Fig. 2).

Table 3 shows the different characteristics of patients with PA according to the method of discovery. PA patients discovered with incidental findings were older (54.8 ± 10.9 vs. 49.1 ± 11.9 years, *P* < 0.001) and less frequently belonged to the female sex (47% vs. 54%, *P* = 0.027) than those discovered with clinical symptoms. The subtypes of PA also differed according to the method of discovery. For PA patients identified incidentally, the distribution between UHA and BHA was similar (46.6% vs. 36.7%). In contrast, among patients diagnosed with clinical symptoms, a higher prevalence of UHA was observed (62.5% vs. 27.4%), indicating a notable difference in PA subtypes based on the method of discovery. However, there was no significant difference in the adenoma size between PA diagnosed with incidental findings and those diagnosed with clinical symptoms. The BP and DDD of antihypertensive drugs were significantly higher in PA patients diagnosed with clinical symptoms (all *P* < 0.001). PAC and the proportion of patients with hypokalemia were higher in PA patients with clinical symptoms (32.2 [23.6;48.1] vs. 26.6 [21.2;36.3], *P* < 0.001 for PAC: 45% vs. 65%, *P* < 0.001 for hypokalemia).

**Table 3:** Comparison of the clinical characteristics of PA patients according to the reason to evaluate for PA

	Incidental findings (*n* = 597)	Clinical symptoms (*n* = 467)	*P*
Age (years)	54.8 ± 10.9	49.1 ± 11.9	**<0.001**
Female (*n*, %)	279 (46.7%)	251 (53.7%)	**0.027**
Height (cm)	164.2 ± 8.3	163.8 ± 8.5	0.497
Weight (kg)	69.8 ± 13.1	68.4 ± 15.1	0.119
BMI (kg/m^2^)	25.8 ± 3.5	25.3 ± 4.2	0.067
SBP (mmHg)	140.0 ± 17.9	145.8 ± 20.4	**<0.001**
DBP (mmHg)	86.0 ± 11.6	91.5 ± 14.1	**<0.001**
Subtype			**<0.001**
UHA	278 (46.6%)	292 (62.5%)	
UHA with ipsilateral nodule	218 (36.5%)	247 (53.0%)	
UHA with contralateral nodule	21 (3.5%)	10 (2.1%)	
UHA with bilateral nodule	23 (3.9%)	22 (4.7%)	
UHA without nodule	16 (2.7%)	13 (2.8%)	
BHA	219 (36.7%)	128 (27.4%)	
BHA with bilateral nodule	22 (3.7%)	7 (1.5%)	
BHA with unilateral nodule	162 (27.1%)	74 (15.9%)	
BHA without nodule	35 (5.9%)	46 (9.9%)	
Indeterminate PA^a^	100 (16.8%)	47 (10.1%)	
Nodule size (cm)	1.6 ± 0.8	1.6 ± 0.8	0.771
PAC (ng/dL)	26.6 [21.2;36.3]	32.2 [23.6;48.1]	**<0.001**
PRA (ng/mL/hr)	0.23 [0.20;0.46]	0.20 [0.10;0.40]	0.336
ARR (ng/dL per ng/mL/h)	103.5 [54.8;190.8]	174.0 [71.1;318.3]	**0.003**
Lowest K (mmol/L)	3.8 [3.2; 4.1]	3.4 [2.9; 3.9]	**<0.001**
Hypokalemia^b^ (*n*,%)	172 (44.9%)	303 (65.0%)	**<0.001**
HTN (*n*, %)	553 (92.6%)	455 (97.4%)	**0.002**
Duration of HTN (years)	7.6 ± 7.7	6.9 ± 7.2	0.123
Antihypertensive drug, DDD	1.9 ± 1.7	2.3 ± 1.9	**0.001**
Antihypertensive drug, DDD ≥ 3 (*n*, %)	152 (26.3%)	147 (31.9%)	0.059
DM (*N*, %)	97 (16.3%)	61 (13.1%)	0.169
CKD ≥ stage 3^c^ (*N*, %)	37 (6.2%)	31 (6.8%)	0.834
CAD^d^ (*N*, %)	44 (7.4%)	39 (8.4%)	0.617
Atrial fibrillation (*N*, %)	9 (1.5%)	6 (1.3%)	0.975
CVA (*N*, %)	38 (6.4%)	32 (6.9%)	0.825

*ARR* aldosterone renin ratio; *BHA*, bilateral hyperaldosteronism; *BMI*, body mass index; *CAD*, coronary artery disease; *CKD*, chronic kidney disease; *CVA*, cerebrovascular disease; *DBP*, diastolic blood pressure; *DM*, diabetes mellitus; *DDD*, daily drug dosage; *HTn*, hypertension; *K*, potassium; *PA*, primary aldosteronism; *PAC*, plasma aldosterone concentration; *PRA*, plasma renin activity; *OSA*, obstructive sleep apnea; *SBP*, systolic blood pressure; *UHA*, unilateral hyperaldosteronism. *P* values < 0.05, indicating statistical significance, are highlighted in bold

Data are shown as a mean ± standard deviation for continuous variables and as *n* (%) for categorical variables. A Student *t*-test was used for continuous variables and a Chi-square test for categorical variables

^a^To determine the PA subtype, we used the lateralization index (LI), which was calculated by dividing the aldosterone to cortisol ratio on the dominant side by that on the non-dominant side. The intermediate subtype was defined as an LI with intermediate values (3 ≤ LI < 4)

^b^Hypokalemia was defined if the serum potassium was <3.5 mEq/L or a patient was taking a potassium supplement

^c^CKD ≥ stage 3 was defined as the estimated glomerular filtration rate (eGFR) < 60 mL/min/1.73 m^2^

^d^CAD was defined if patients had a history of percutaneous coronary intervention (PCI), coronary artery bypass graft (CABG) surgery, or unstable angina

**Fig. 3:**
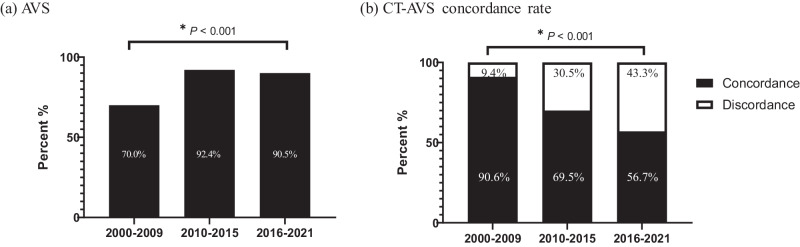
Temporal trend of CT-AVS concordance rate in patients with primary aldosteronism. Temporal trend of **a** the percentage of patients who performed AVS and **b** CT-AVS concordance rate in patients with primary aldosteronism. CT computed tomography, AVS adrenal venous sampling

The concordance rate between CT and AVS has changed over the last 20 years. From time period 1 to 2, the proportion of patients undergoing AVS increased dramatically (70.0% to 92.4%, *P* < 0.001) (Fig. 3a). The concordance rate between CT and AVS significantly declined over time (90.6%, 69.5%, and 56.7%, *P* for trend <0.001) (Fig. 3b).

## Discussion

In this large cohort study involving 1064 PA patients observed for over 20 years, we found that the clinical and biochemical severity of PA attenuated, and the number of patients with BHA increased from 2000 to 2021. In the subtype-specific analysis, each subtype of PA tended to become milder, particularly BHA. PA was more commonly discovered incidentally on imaging, and the discordance rate between CT and AVS increased over the last 20 years.

The severity of PA tended to decrease while the number of PA patients increased. Our results spanning 20 years of data are consistent with those of a multicenter retrospective study of 710 PA patients from the German Conn’s Registry, which also reported a trend of milder subtypes of PA within 9 years [11]. Why the severity of PA decreased remains uncertain, but it may be attributed mostly to changes in the proportion of subtypes of PA. In the present study, while the incidence of UHA declined, BHA incidence increased from 2000 to 2021; this finding is consistent with the results of studies using the Korean [12] and German Conn’s Registry [11]. The increased incidence of BHA, which usually has milder severity than UHA [9, 18], might have contributed to the milder severity of PA in recent years. The increased frequency of screening tests, attributed to the widespread recognition of PA as a leading cause of secondary HTN, along with the increased incidental detection of adrenal nodules, could be associated with the increased incidence of BHA in recent years [11, 12].

However, in contrast to our findings regarding the decreasing trend of the clinical severity, including the prevalence of hypokalemia in both UHA and BHA subtypes, a Japanese study showed a trend toward milder severity associated with the duration of HTN and prevalence of hypokalemia in the BAH subtype but not in the APA subtype [19]. In terms of the PA clinical severity trends we have here observed, the decreasing trend of the biochemical severity including decreased ARR in both UHA and BHA subtypes, and decreased PAC in the BHA subtype, were not reported in the Japanese study [19]. The difference in temporal trend severity between subtypes remains unknown. The more severe phenotype in the present study by adding the criteria of PAC > 15 ng/dL to ARR ≥20 for the screening test and various confirmatory tests in the Japanese study [19] might be the reason.

Despite the decreasing trend of ARR in both UHA and BHA, the increasing tendency of PRA and decreasing tendency of PAC were only in UHA and BHA, respectively. Although the cause of differences in trends of PRA and PAC between UHA and BHA was uncertain, early detection through the workup for increased incidental detection of adrenal nodules might be the reason. PRA was persistently low despite the normalized PAC at 3 months after adrenalectomy [20], so early detection in patients with UHA might affect the degree of suppressed PRA but not severe aldosterone excess. Conversely, early detection in PA patients with BHA might affect the mild-to-moderate aldosterone excess rather than the degree of suppressed PRA.

Despite the mildness of PA in recent years, the prevalence of comorbidities such as DM, CKD, CAD, atrial fibrillation, and CVA did not differ across time periods. However, Murata et al. indicated that patients with mild PA did not show a high cardiovascular risk when compared to those with essential hypertension [21]. Due to the cross-sectional design of our study, the association between milder PA and relatively lower cardiovascular risk could not be determined. Whether milder forms of PA have a cardiovascular risk similar to that of essential hypertension remains to be elucidated.

The proportion of PA patients diagnosed following incidental findings was increased while that of cases diagnosed based on clinical symptoms had declined over the study period. The recently increased use of CT imaging for health check-ups has led to this increased incidental detection of adrenal nodules, further encouraging the performance of screening tests for PA. In addition, the higher incidence of incidentally detected PA in men compared to women may be related to the higher rate of health screening participation among men [22]. Our current PA patients with incidental findings showed a milder form of the disease than those with clinical symptoms. Therefore, changes in the method of discovery also might affect the clinical and biochemical characteristics of PA in the present study. Few studies have compared the characteristics of patients with PA according to the method of discovery. In contrast to our findings, milder severity in PA patients with incidental findings than those with clinical symptoms, Kuo et al. reported that there was no significant difference in the clinical and biochemical characteristics according to the method of discovery only in PA patients who underwent adrenalectomy [14]. However, PA patients who underwent adrenalectomy had moderate-to-severe APA. Therefore, various severity and subtypes of PA patients in our study might contribute to the differences in characteristics of PA patients according to the method of discovery.

The rate of AVS implementation showed an increasing trend from 70.0% in time period 1 to over 90% in time periods 2 and 3. This increase was attributed to a reluctance to perform AVS during time period 1 due to technical issues, and to the high number of cases where surgery was performed without AVS on patients who were young, had a unilateral adrenal mass, exhibited marked hyperaldosteronism, or had hypokalemia. Few studies have examined the secular trends in the concordance rate between CT and AVS. In our study of a large number of patients with successful AVS results, the overall concordance rate between CT and AVS findings was 64.4%, similar to 64% in the previous study [15]. The concordance rate of CT-AVS findings dramatically declined over the 20 years from 90.6% in time period 1 to 56.7% in time period 3. This may be attributed to an increase in the number of PA patients with incidental findings. We noticed a higher proportion of BHA with unilateral adrenal nodules and UHA with contralateral nodules over 20 years, which indicates CT-AVS discordance. The role of AVS in subtyping should be emphasized due to the higher discordance rate between CT and AVS findings. AVS under ACTH stimulation could induce misclassification of mild APA as BAH with unilateral adrenal nodule despite improved successful catheterization [19, 23].

The German study showed a trend toward an increasing proportion of female PA patients in recent years [11]. In contrast, in the Japan study, no sex-related differences were found over time, although the proportion of females was higher among BAH patients than among APA patients at the time of diagnosis [19]. We also found an increased proportion of females only in BHA patients in time period 3. However, among all patients with PA, the ratio of males to females was similar across the three different time groups. These discrepant findings cannot be fully explained, but they pertain to sex and ethnic differences in the genetic etiology of PA. In Asia, the main mutated driver gene in APA cases is the KCNJ5 gene, which is also prevalent in females and is characterized by more severe clinical features [24]. Therefore, in Asia, the detection of APA harboring *KCNJ5* gene mutation might not show temporal trends. This also explains the female preponderance among PA patients with clinical symptoms rather than those discovered incidentally.

Our study has several strengths. First, compared to the German study and the Japanese study which showed a time trend of PA over only 9 and 13 years, the present study revealed a long-term trend of PA over 20 years. Second, we enrolled a relatively large number of cases, exceeding 1000, thus ensuring a comprehensive study sample. Third, we compared the clinical and biochemical characteristics of patients with PA according to the method of discovery. Last, our study is the first study to explore the temporal trends of the CT-AVS concordance rate, which has been known to be around 64% [15].

Nevertheless, our study also has several limitations. First, there were only 129 PA patients in period 1 (2000–2009), which is a relatively small population compared to that in the other two-time period groups. Second, because we enrolled patients from two tertiary hospitals, the results cannot be generalized to other centers or ethnicities. Third, there may be some differences in the protocol for screening and diagnosing PA between the two tertiary hospitals in which we enrolled patients. However, the AVS process was similar in terms of sequential sampling under ACTH infusion. Fourth, in this study, PRA and PAC were measured using an RIA kit without LC-MS/MS standardization. Last, this study investigated the temporal trends in PA over more than 20 years and primarily included patients treated before the widespread implementation of techniques such as CYP11B2 immunohistochemistry. Consequently, our findings do not reflect the histopathological diagnosis criteria of the 2022 WHO classification of adrenal cortical tumors.

## Asian perspectives

Differences in gene variants between European and Asian populations, such as the higher prevalence of *KCNJ5* mutation in Asia [24], could influence the temporal trends of PA, including the male-to-female ratio. Additional exploration is needed on the impact of these genetic differences on the trends of PA.

## Conclusion

In conclusion, the proportion of patients with PA discovered incidentally, and BHA has increased over the past 20 years. With these changes, more patients are likely to present with milder clinical symptoms and biochemical profiles. Since the concordance rate between CT and AVS findings has declined, more caution is required when interpreting CT results in patients with PA. Whether early detection of mild PA results in better outcomes remains to be elucidated.

### Supplementary information

Supplementary Table S1
