# Supplementary material for: Temporal trends in clinical features of patients with primary aldosteronism over 20 years
Source: Hypertens Res. 2024 May 17;47(8):2019–28. doi: 10.1038/s41440-024-01703-w (PMC11298405; doi:10.1038/s41440-024-01703-w)
Supplement: Supplementary file 1 — Supplementary Table S1 [file 41440_2024_1703_MOESM1_ESM.docx]

Supplementary Table S1. Trends in the clinical characteristics of PA patients according to the period of diagnosis

|  | 2000-2007  (n=78) | 2008-2014  (n=276) | 2015-2021  (n=710) | Total  (n=1,064) | *P* for trend |
| --- | --- | --- | --- | --- | --- |
| Age (years) | 47.7 ± 11.5 | 52.9 ± 11.4 | 52.6 ± 11.7 | 52.3 ± 11.7 | 0.101 |
| Female (n, %) | 42 (53.8%) | 127 (46.0%) | 361 (50.8%) | 530 (49.8%) | 0.680 |
| Height (cm) | 162.9 ± 8.6 | 163.5 ± 8.1 | 164.4 ± 8.5 | 164.0 ± 8.4 | 0.074 |
| Weight (kg) | 64.8 ± 11.0 | 67.6 ± 12.9 | 70.3 ± 14.6 | 69.2 ± 14.0 | 0.001 |
| BMI (kg/m2) | 24.2 ± 3.0 | 25.1 ± 3.5 | 25.9 ± 4.0 | 25.6 ± 3.8 | <0.001 |
| SBP (mmHg) | 153.7 ± 25.6 | 142.1 ± 19.6 | 141.4 ± 17.9 | 142.5 ± 19.3 | 0.040 |
| DBP (mmHg) | 97.1 ± 18.6 | 88.1 ± 13.5 | 87.6 ± 11.7 | 88.4 ± 13.0 | 0.005 |
| Subtype |  |  |  |  |  |
| UHA | 61 (78.2%) | 180 (65.2%) | 329 (46.3%) | 570 (53.6%) | <0.001 |
| BHA | 7 (9.0%) | 64 (23.2%) | 276 (38.9%) | 347 (32.6%) | <0.001 |
| Indeterminate PA^a^ | 10 (12.8%) | 32 (11.6%) | 105 (14.8%) | 147 (13.8%) | 0.273 |
| Nodule size (cm) | 1.6 ± 0.5 | 1.6 ± 0.8 | 1.6 ± 0.8 | 1.6 ± 0.8 | 0.565 |
| PAC (ng/dL) | 37.8 [27.8;55.3] | 30.8 [22.6;46.3] | 26.9 [21.2;38.2] | 28.8 [21.9;41.5] | <0.001 |
| PRA (ng/mL/hr) | 0.1 [ 0.1; 0.4] | 0.2 [ 0.1; 0.3] | 0.2 [ 0.2; 0.5] | 0.20 [0.10;0.45] | <0.001 |
| ARR (ng/dL per ng/mL/h) | 242.5 [84.5;389.0] | 162.8 [76.6;365.0] | 106.0 [54.7;204.6] | 119.0 [59.3;251.4] | <0.001 |
| Lowest K (mmol/L) | 3.0 [ 2.7; 3.3] | 3.4 [ 2.9; 3.9] | 3.8 [ 3.2; 4.1] | 3.6 [ 3.0; 4.0] | <0.001 |
| Hypokalemia^b^ (n, %) | 68 (87.2%) | 185 (67.5%) | 291 (41.0%) | 544 (51.3%) | <0.001 |
| HTN (n, %) | 78 (100.0%) | 271 (98.2%) | 659 (92.8%) | 1008 (94.7%) | <0.001 |
| Duration of HTN (years) | 6.2 ± 6.3 | 7.4 ± 6.5 | 7.3 ± 8.0 | 7.3 ± 7.5 | 0.319 |
| Antihypertensive drug, DDD | 1.9 ± 1.6 | 2.2 ± 1.7 | 23.0 ± 168.5 | 2.1 ± 1.8 | 0.523 |
| Antihypertensive drug, DDD ≥ 3 (n, %) | 17 (22.1%) | 81 (30.3%) | 201 (29.0%) | 299 (28.8%) | 0.491 |
| DM (n, %) | 9 (11.5%) | 34 (12.3%) | 115 (16.2%) | 158 (14.9%) | 0.091 |
| CKD ≥ stage 3^c^ (n, %) | 7 (9.0%) | 20 (7.4%) | 41 (5.8%) | 68 (6.5%) | 0.199 |
| CAD^d^ (n, %) | 4 (5.1%) | 25 (9.1%) | 54 (7.6%) | 83 (7.8%) | 0.880 |
| Atrial fibrillation (n, %) | 3 (3.8%) | 3 (1.1%) | 9 (1.3%) | 15 (1.4%) | 0.228 |
| CVA (n, %) | 5 (6.4%) | 26 (9.4%) | 39 (5.5%) | 70 (6.6%) | 0.137 |

ARR, aldosterone renin ratio; BHA, bilateral hyperaldosteronism; BMI, body mass index; CAD, coronary artery disease; CKD, chronic kidney disease; CVA, cerebrovascular disease; DBP, diastolic blood pressure; DM, diabetes mellitus; DDD, daily drug dosage; HTn, hypertension; K, potassium; PA, primary aldosteronism; PAC, plasma aldosterone concentration; PRA, plasma renin activity; OSA, obstructive sleep apnea; SBP, systolic blood pressure; UHA, unilateral hyperaldosteronism.

Data are shown as mean ± standard deviation for continuous variables, n (%) for categorical variables. A Jonckheere-Terpstra trend test was used for continuous variables and a Cochran-Armitage trend test for categorical variables.

^a^To determine the PA subtype, we used the lateralization index (LI), which was calculated by dividing aldosterone to cortisol ratio on the dominant side by that on the non-dominant side. The intermediate subtype was defined as an LI with intermediate values (3≤ LI <4).

^b^Hypokalemia was defined if the serum potassium was <3.5 mEq/L or a patient was taking a potassium supplement.

^c^CKD ≥ stage 3 was defined as the estimated glomerular filtration rate (eGFR) < 60 mL/min/1.73 m2.

^d^CAD was defined if patients had a history of percutaneous coronary intervention (PCI), coronary artery bypass graft (CABG) surgery, or unstable angina.
